# Exploring the application of Artificial Intelligence in palliative care and its practical, technical and ethical considerations: a scoping review

**DOI:** 10.1186/s12904-026-02191-0

**Published:** 2026-06-29

**Authors:** Thanarpan Peerawong, Tharin Phenwan, Sitthichok Chaichulee, Kamonrat Tangudomkit

**Affiliations:** 1 Faculty of Medicine, Prince of Songkla University, Songkhla, Thailand; 2 Faculty of Health, University of Dundee, Dundee, UK

**Keywords:** Digital health technologies, Artificial Intelligence, Machine learning, Digital tools, Scoping review

## Abstract

**Background:**

Palliative care improves the quality of life of people living with life-limiting conditions and their families; however, global access remains constrained by workforce shortages and late referrals. Artificial Intelligence (AI) has been proposed as a scalable solution for optimising the identification of needs, supporting clinical decision-making, and enhancing care delivery. However, real-world evidence of the application of AI in palliative care remains sparse, particularly regarding its impact on quality of life, quality of care, and associated practical, technical and ethical challenges.

**Methods:**

A scoping review was conducted following the Joanna Briggs Institute methodology and the PRISMA-ScR checklist. Five databases (the ACM Digital Library, CINAHL, Cochrane Central, PubMed and Web of Science) were searched between September and October 2025. Studies reporting the use of AI to facilitate or enhance palliative care delivery in adults were eligible. Four reviewers independently screened the records, and two reviewers extracted the data using Covidence software. A narrative synthesis was then performed.

**Results:**

Fifteen studies, published between 2021 and 2025, were included. Fourteen originated from Global North settings (USA 5, Germany 2, Japan 2, Taiwan 2, UK 1, Spain 1 and Cyprus 1) and one from Iran. Conceptually, AI applications fall into three domains: (1) early identification of palliative care needs, (2) symptom assessment and management and (3) clinical decision support for care conversations. Fifteen studies (100%) reported or discussed quality of care outcomes, most commonly prognostic performance, usability and referral/conversation rates, and only two (13.3%) directly addressed quality of life. Effectiveness was consistently positive, with four randomised controlled trials demonstrating superiority over usual care in referrals, advance care planning, pain control and quality of life domains. Practical barriers were centred on workflow integration and resource demands, while technical limitations include data quality, generalisability, and interpretability. Ethical discourse is underdeveloped, with major gaps in the principles of AI governance.

**Conclusions:**

AI shows potential to improve prognostic accuracy, trigger earlier involvement of palliative care specialists and support symptom management. However, this evidence is geographically skewed, methodologically immature and ethically underdeveloped. Future research must prioritise diverse global settings, patient-reported quality of life outcomes, participatory co-design and systematic ethical governance to ensure equitable implementation.

**Supplementary Information:**

The online version contains supplementary material available at https://doi.org/10.1186/s12904-026-02191-0.

## Background

Palliative care improves the quality of life of people living with life-limiting conditions and their families by addressing their physical, psychological, social and spiritual needs [1]. Early delivery of palliative care has been shown to reduce symptom burden, decrease unwanted aggressive interventions at the end of life and improve both people receiving palliative care’ and carers’ outcomes [2, 3]. Despite these benefits, access remains limited worldwide, particularly due to workforce shortages, late referrals and resource constraints in both high- and low-income settings [4, 5].

Artificial intelligence (AI) and related technologies have emerged as potential tools to address these gaps [6–8]. AI systems can analyse electronic health records, wearable sensor data or conversational voice data to identify unmet palliative care needs, predict prognosis, support symptom monitoring and facilitate conversations about serious illnesses [7, 8]. However, evidence of real-world applications in palliative care remains limited, with most studies focusing on retrospective electronic health record data rather than prospective use with people receiving palliative care or caregivers in diverse clinical contexts.

Few studies have evaluated the impact of AI on core palliative care outcomes, including patient quality of life (QoL) and quality of care (QoC). Moreover, although recent reviews have highlighted practical, technical and ethical challenges, comprehensive mapping is lacking [7–9]. This is particularly concerning, given the vulnerability of the population and the additional ethical considerations that arise when AI interfaces directly with human decision-making in end-of-life care [10–13].

With AI gaining rapid global momentum in healthcare, there is an urgent need to systematically capture the emerging evidence of its role in palliative care delivery, its effectiveness in improving QoL and QoC, and its associated practical, technical and ethical implications. Of particular concern is the risk that AI, developed predominantly in high-resource settings, may exacerbate existing inequities when applied globally. Therefore, this scoping review aims to:

1. map existing evidence on the application of AI in palliative care in a real-world context.
2. examine its reported effectiveness and influence on the quality of life and quality of care.
3. identify implicit and explicit practical, technical and ethical considerations, together with proposed mitigation strategies.

### Review questions

1. What types of AI have been implemented to improve palliative care delivery?
2. Which health-related outcomes in the QoL and QoC domains have been measured or discussed?
3. How effective are these AI applications in relation to the measured or discussed outcomes?
4. What practical, technical and ethical considerations have been identified, and what mitigation strategies have been proposed?

For this review, relevant definitions from the United Kingdom Research Integrity Office (UKRIO) were used [14] p2:

- AI is an umbrella term for a range of algorithm-based technologies that solve complex tasks by carrying out functions that previously required human thinking.
- Machine learning (ML) is a subset of AI that learns from data without being explicitly programmed. This learning can be unsupervised or human instructed/’human-in-the-loop’ (reinforcement learning)’.
- Natural language processing (NLP) refers to processes that enable machines to understand, generate and manipulate human language.
- Generative AI is an ‘AI that can create new text, images, audio, video, and code’. An example includes ChatGPT, which is ‘a large language model (LLM) designed to receive text cues or prompts (inputs) and generate outputs using NLP.
- Multimodal AI refers to the use of multiple AI systems to process different types of data simultaneously.

## Methods

A scoping review was conducted following the Joanna Briggs Institute (JBI) methodology for scoping reviews and reported according to the Preferred Reporting Items for Systematic Reviews and Meta-Analyses extension for Scoping Reviews checklist [15]. The review protocol was registered in the Open Science Framework platform [16].

### Eligibility criteria

Studies were included based on inclusion and exclusion criteria (Table 1). The Population, Concept and Context framework was used to formulate the criteria and review questions [17].

**Table 1:** Inclusion and exclusion criteria

	Inclusion criteria	Exclusion criteria
Population	Articles that discuss: - Adults receiving palliative care - Family carers of adults receiving palliative care - Healthcare professionals delivering palliative care	- Children
Concept	- Any application on real-world/contemporaneous use of AI to facilitate or enhance palliative care delivery (e.g., prognostication, symptom management, decision support, communication)	- Articles not related to palliative care provision - Theoretical discussions, legal/ethical/theoretical debates without any application
Context	- Any context of care (acute hospitals, long-term care facilities, community) - Global	–
Types of Evidence	- Quantitative studies - Qualitative studies - Mixed methods - Grey literature - Published in English	- Reviews, editorials, protocols, theses - Articles published in other languages

### Search strategy

Four team members, supported by an academic librarian, searched the Association for Computing Machinery Digital Library, the Cumulative Index to Nursing and Allied Health Literature, Cochrane Central, PubMed and Web of Science databases between September and October 2025. A combination of keywords, Boolean operators and truncations was applied to maximise the retrieval of relevant studies (see Supplementary File 1). Grey literature was eligible; however, searches were limited to the databases listed above.

### Screening and selection

The search results were uploaded to Covidence, a review management software programme for semi-automated duplicate removal. Duplicates were further removed manually.

Initially, four reviewers performed a 25-title pilot testing against the inclusion and exclusion criteria and discussed any ambiguities. Subsequently, a dual-screening process was applied. Four reviewers were randomly assigned to screen the articles independently. The lead reviewer was responsible for the final decision on whether to include or exclude records in cases of discrepancy. Titles with unclear or absent abstracts were included in the full-text review.

A similar process was then applied to the full-text review, starting with a pilot testing against the inclusion and exclusion criteria, followed by a dual-screening process of randomly assigned articles, with the lead reviewer resolving discrepancies. The reasons for exclusion were documented at the full-text stage.

### Data extraction and synthesis

A pilot extraction file was created using the Covidence software. The lead reviewer then extracted the relevant data from the pilot extraction file, and the process was iterative, ensuring that the review questions were fully answered. Once finalised through team discussions, two reviewers extracted the remaining data until completion. The remaining reviewers verified the completeness of the extracted data.

The extracted data included:

*Author(s); Year of publication; Country of origin; Aims/objectives; Population and sample size; Concept; Methodology/methods used; Context of the study; Types of AI used; AI development and testing process, if relevant; Outcome measures/discussed; Influences/effects of AI used on quality of life and quality of care; Practical considerations; Technical considerations; Ethical considerations.*

Study authors were not contacted for clarification or to obtain missing data.

No formal critical appraisal of individual study quality was undertaken, which is consistent with the JBI scoping review guidelines [17].

### Synthesis of results

Due to the anticipated heterogeneity in study designs and outcomes, findings were narratively synthesised and presented in tables and figures [18]. The included studies were categorised according to their shared characteristics, and the findings were related to the review questions.

### Patient and public involvement

There was no direct patient or public involvement in this review.

## Results

### Overall characteristics of the included studies

**Fig. 1:**
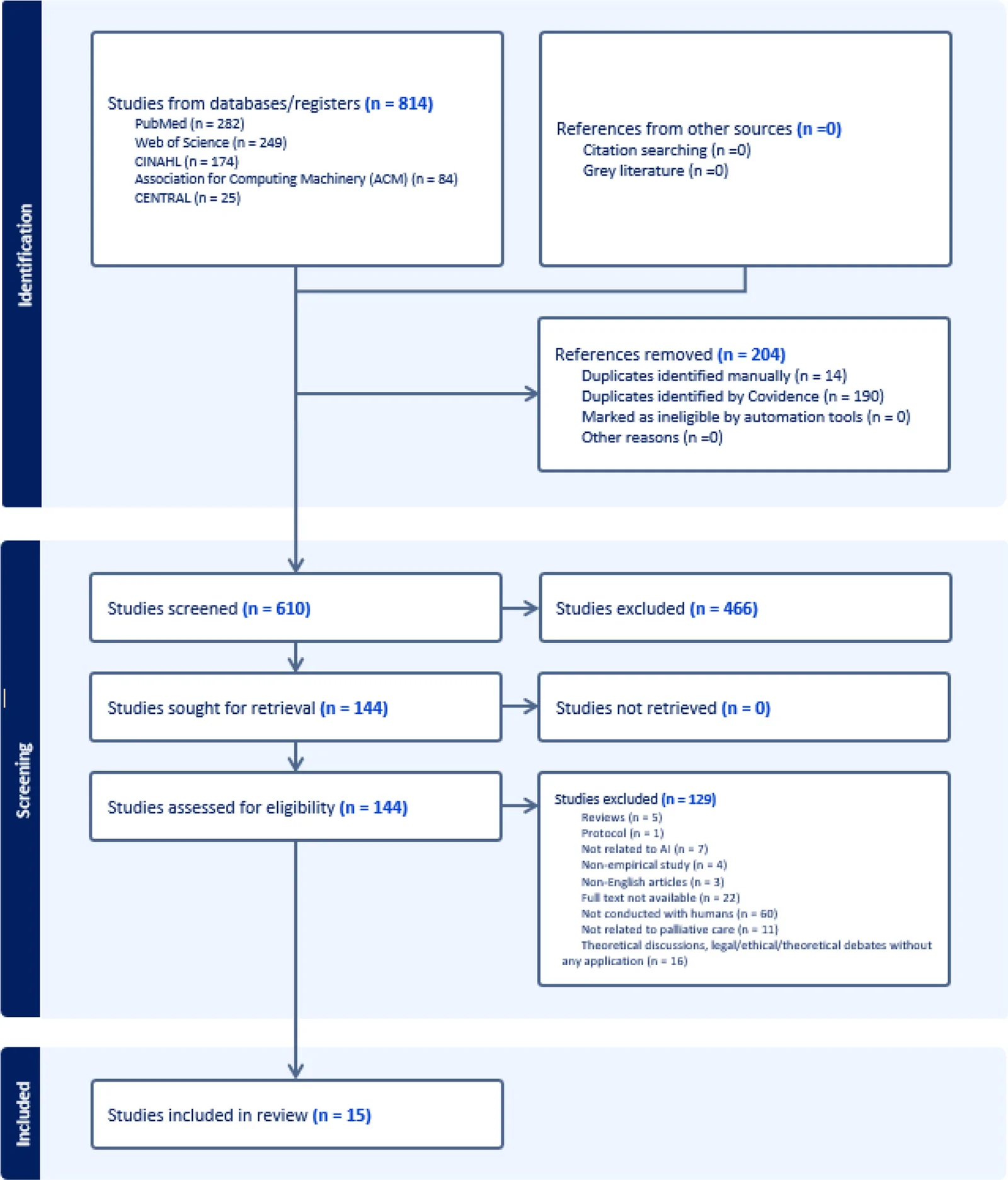
PRISMA flow diagram

The databases were searched between September and October 2025. In total, 814 records were identified. Of these, 204 duplicates were removed, and 610 titles and abstracts were screened. In total, 144 articles were included in the full-text assessment. Of these, 129 articles were excluded, leaving 15 studies for review (see Fig. 1).

### Study characteristics

The included 15 studies were published between 2021 and 2025 and spanned multiple countries: United states of America [19–23] (*N* = 5); Germany [24, 25] (*N* = 2); Japan [26, 27] (*N* = 2); Taiwan, China [28, 29] (*N* = 2); Cyprus [30] (*N* = 1); Iran [31] (*N* = 1); UK [32] (*N* = 1), and one study conducted in six countries [33] (Brazil, Italy, Greece, Portugal, Scotland and Spain), indicating global interest in the topic.

Only one study originated from the Global South (Iran) [31].

Most studies were conducted in acute hospitals [20–26, 28, 29, 31, 32] (*N* = 11), followed by multiple settings [30, 33] (*N* = 2) and community settings [19, 27] (*N* = 2). No studies focused on people receiving palliative care admitted to long-term care facilities.

Regarding study designs, 13 studies employed quantitative designs with only one study utilising a qualitative design [19]. One study used a quasi-experimental design [20]. Four studies were randomised controlled trials (RCTs) [21–23, 30]. Two studies used a prospective cohort design [28, 29], while one study used a retrospective cohort design [26]. Other designs included vignette-based evaluations [24, 33], cross-sectional studies [27, 32], device validation studies [25], observational predictive modelling studies [31], and qualitative focus group studies [19]. None utilised a participatory design.

In terms of conditions, six studies focused on advanced cancer [20, 22, 26, 28–30] and one study focused on any stage of cancer [21]. One study included people with unspecified palliative conditions [25]. One study included vignettes of people living with gynaecological cancer [24], while another focused on vignettes of people receiving palliative care with a poor prognosis [33]. Two studies focused on multiple palliative conditions [19, 23, 27], one on chronic obstructive pulmonary disease (COPD) [31], and one on people with dementia [32].

The sample sizes of the included studies ranged from 3 to 20,506 (median 112). Twelve studies primarily involved people receiving palliative care (with or without family carers as secondary participants) or used palliative care records [20–23, 25–32]. Three studies focused primarily on healthcare professionals (HCPs) as the end users of AI tools [19, 24, 33] (See Table 2).

**Table 2:** Characteristics of the included studies

Study	Country	Design	Participants	Context	Conditions
Bowles et al. [19]	United States of America (USA)	Qualitative study using focus group interviews	3 palliative care team members	Home and community health provider	3 seriously ill people receiving palliative care as identified by the algorithm
Gensheimer et al. [20]	USA	Quantitative interventional study with a controlled before-and-after design	1,251 people receiving palliative care with metastatic cancer	Acute hospital, 4 oncology clinics	Metastatic cancer
Manz et al. [21]	USA	Randomised controlled trials (RCT)	20,506 cancer people receiving palliative care (41,021 encounters)	Acute hospital, 9 tertiary and community-based oncology clinics	Cancer (any stage)
Kamdar et al. [22]	USA	RCT	112 people receiving palliative care with advanced cancer	Acute hospital, outpatient palliative care clinic	Advanced cancer
Wilson et al. [23]	USA	RCT	2,544 people receiving palliative care	Acute hospitals, 12 nursing units	Multiple medical conditions
Braun et al. [24]	Germany	Quantitative descriptive study with expert evaluation of case vignettes	5 experts in gynaecologic oncology (3) and palliative care (2)	Unspecified, implied academic medical centres	10 patient vignettes of gynaecologic cancers with metastases receiving palliative care
Griebhammer et al. [25]	Germany	Quantitative comparative clinical trial	34 and 19 palliative care people receiving palliative care in study arms I and II, respectively	Acute hospital	Life-threatening illnesses requiring hospitalisation
Kawashima et al. [26]	Japan	Quantitative, retrospective cohort study	561 people receiving palliative care with advanced cancer	Acute hospital	Advanced cancer receiving chemotherapy
Dong et al. [27]	Japan	Quantitative, cross-sectional study	100 home-based palliative care people receiving palliative care	Community	Multiple medical conditions
Yang et al. [28]	Taiwan	Quantitative, prospective observational study	60 end-stage cancer people receiving palliative care	Acute hospital	End-stage cancer
Liu et al. [29]	Taiwan	Quantitative, prospective observational cohort study	40 terminal cancer people receiving palliative care	Acute hospital	Terminal cancer
Haleem et al. [30]	Cyprus	Quantitative RCT with a between-subject design	999 people receiving palliative care with advanced cancer	Community-based cancer care organisation	Advanced cancer
Nejatifar et al. [31]	Iran	Quantitative, cross-sectional study	140 people receiving palliative care with chronic obstructive pulmonary disease (COPD)	Acute hospital, respiratory clinic	COPD with frailty indicators
Sampson et al. [32]	United Kingdom	Quantitative, cross-sectional study	63 people with moderate and severe dementia	Acute hospitals, 6 wards	Dementia
Blanes-Selva et al. [33]	Italy, Brazil, Spain, Greece, Scotland, Portugal	Mixed-methods study	21 palliative care healthcare professionals (HCPs), including 15 physicians and 6 nurses	Multiple healthcare settings where HCPs use a web-based palliative care clinical decision support system	6 patient vignettes with a poor prognosis

### How AI is used

Three types of AI have been conceptually identified, with some studies focusing on more than one category.

#### Early identification of palliative care needs (*N* = 9)

This concept is the dominant application, present in almost every year of publication and is particularly prominent from 2021 to 2023. These studies used ML or deep learning (DL) models applied to electronic health records, claims data, frailty indices or wearable actigraphy to predict mortality, deterioration or specialist palliative care requirements, with the aim of triggering earlier referral or advance care planning [19–21, 23, 26, 28, 29, 31, 33].

#### Symptoms assessment and management (*N* = 6)

This concept became more prominent between 2024 and 2025, reflecting the emergence of novel, multimodal and sensor-based approaches. These include AI-based facial recognition for pain assessment in people with dementia [32], contactless radar heart-rate monitoring [25], voice recognition for patient-reported outcome extraction [27], multimodal deep-learning QoL estimation from wearables [30], digital therapeutic applications with AI triage for cancer pain [22], and evaluation of ChatGPT therapy suggestions [24].

#### Clinical decision support for care conversations (*N* = 4)

This concept appeared consistently but less frequently across the period, often combining predictive models with behavioural nudges [20, 21], generative LLMs to translate claims data into narrative summaries [19], or user-centred clinical decision support systems with explainability features [33].

Some studies contributed to more than one concept. For instance, Haleem (2025) combined multimodal wearable data with patient-reported outcomes to estimate deep-learning QoL. This illustrates the growing convergence of predictive and symptom monitoring capabilities in newer applications (See Table 3).

**Table 3:** Concepts identified and how AI is used

Concepts (N)	Primary AI used	How AI is used	Studies
Early identification of palliative care needs (9)	- Gradient boosting models (XGBoost/GBM) - Machine learning (ML) risk prediction models - Deep learning (DL) sequence models (LSTM/BiLSTM) - Ensemble learning (Super Learner)	- Predict mortality/survival risk (short- to long- term horizons) - Identify frailty or palliative-care needs - Trigger referrals/serious illness conversations - Prioritise advance-care planning	[19–21, 23, 26, 28, 29, 31, 33]
Symptoms assessment and management (6)	- Generative large language models (LLMs) - Multimodal DL - Facial recognition ML - Radar-based ML - Voice recognition ML	- Real-time pain/symptom triage - Contactless vital-sign monitoring - Automated patient-reported outcome measures extraction from voice - Multimodal quality-of-life estimation from wearables - Generate/evaluate symptom-management advice	[22, 24, 25, 27, 30, 32]
Clinical decision support for care conversations (4)	- Generative LLMs - Behavioural nudges - SHapley Additive exPlanations explainability - User-centred clinical decision support systems (CDSS)	- Translate claims/electronic health record data into narrative summaries - Trigger nudges (emails/lists) for conversations - Present predictions vs. clinician judgement in CDSS - Support shared decision-making	[19–21, 33]

**Fig. 2:**
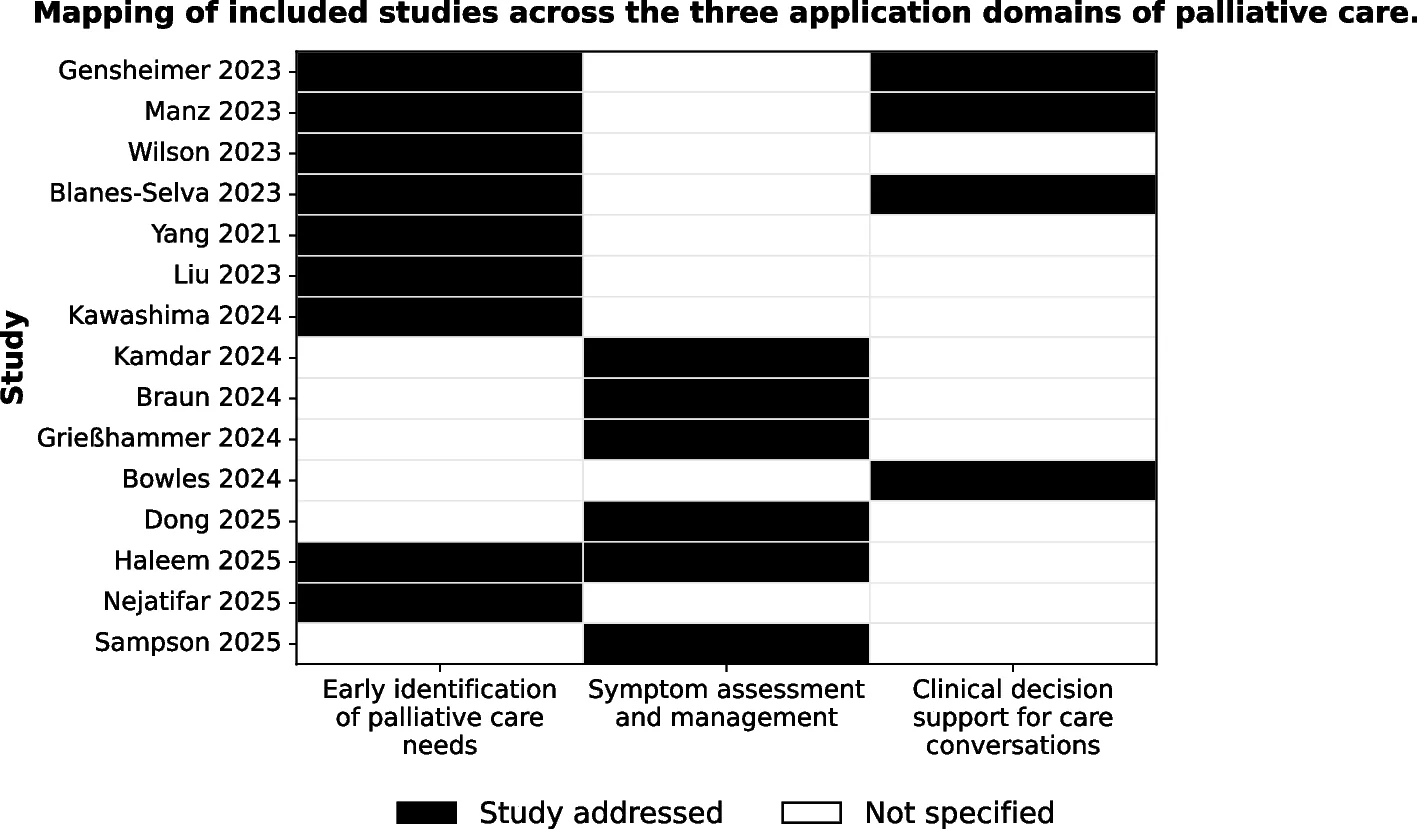
Heatmap of artificial intelligence (AI) applications across palliative-care domains

Figure 2 demonstrates how AI has been applied across palliative care domains.

### AI algorithms and modalities

Structure-data predictive modelling was used in seven studies [20, 21, 23, 26, 29, 31, 33]. These approaches use structured data to train prognostic classifiers (such as gradient boosting, tree-based models and ensemble approaches) to identify people receiving palliative care at higher risk of mortality or requiring specialist palliative input. Most studies have focused on algorithm development and validation [26, 29, 31, 33], with only a few evaluating models integrated into care delivery [20, 21, 23].

Time series and multimodal DL were employed in three studies [28–30], primarily to handle continuous or high-dimensional data. This includes the use of Recurrent Neural Networks and Long Short-Term Memory networks to predict survival outcomes in end-stage cancer [28] and estimate health-related quality of life (HRQoL) from wearable physiology [30].

AI-enabled digital therapeutic applications have been reported in one study [22]. The AI component functions as an interactive decision-support logic to guide tailored self-management content and escalation pathways for pain control.

Generative AI and LLMs were incorporated in two studies [19, 24]. These were applied to unstructured text tasks, specifically generating narrative clinical summaries to support palliative care needs assessment [19] and to evaluate guideline conformity and the quality of therapy suggestions in a palliative setting [24].

Three studies used specialised technologies to facilitate symptom assessment [25, 27, 32]. These include facial recognition to support pain assessment in individuals with dementia [32], voice recognition to detect patient-reported outcomes using conversational audio [27] and contactless radar-based heart rate monitoring as a low-burden physiological modality [25].

### Reported health outcomes

#### Quality of life

In this review, QoL is defined as a patient-reported, multidimensional construct that encompasses an individual’s symptoms, physical and psychological function, and perceived health status [34]. Two of the 15 studies (13%) directly measured QoL using dedicated instrument [22, 30]. Both studies discussed AI as a potential tool for improving the QoL [22, 30]. Although QoL is rarely a primary endpoint, several studies have incorporated QoL-related measures within modelling [26–28], but not as direct QoL outcomes: Kawashima (2024) and Kamdar (2024) used a patient-reported Pain Numerical Rating Scale (NRS) and Pain intensity score (0–10) for modelling specialist palliative-care needs [26], Dong (2024) used Integrated Palliative Care Outcome Scale (IPOS) in their Patient-Reported Outcome Measures (PROM) framework [27], and Yang (2021) used Karnofsky Performance Status (KPS) and Palliative Prognostic Index (PPI) for survival prediction [28].

Additionally, many studies on QoC outcomes have addressed the potential for improving QoL. Kawashima (2024) concluded that earlier identification of palliative-care needs via ML "has the potential to contribute to earlier palliative care" and thereby improve QoL whereas Bowles (2024) briefly mentions in the abstract that the narratives created from generative AI have "the potential to support clinical decision-making" and "improve quality of life" by enabling timelier needs assessment.

#### Patient-level (QoL-related) measures identified across the included studies:

- Functional Assessment of Cancer Therapy General (Kamdar 2024) [22]
- Generalized Anxiety Disorder (Kamdar 2024) [22]
- Brief Pain Inventory (Kamdar 2024) [22]
- HRQoL (Haleem 2025) [30]
- EORTC Quality of Life Questionnaire Core-30 (EORTC HRQoL C30) (Haleem 2025) [30]
- Hospital Anxiety and Depression Scale (Haleem 2025) [30]
- IPOS (Dong 2024, Haleem 2025) [27, 30].
- KPS (Yang 2021) [28]
- Pain NRS (Kawashima 2024) [26]
- Palliative Prognostic Index (Yang 2021) [28] (*clinician-rated patient-level assessments but categorized as QoL on the basis that they describe the individual patient's functional status or prognosis.)*

#### Quality of care

In this review, QoC is treated as a system-, provider-, or tool-level construct that describes how care is structured and delivered [35]. All 15 studies (100%) reported QoC-related outcomes [19–33]. Of these, 14 measured it quantitatively [20–33] and one was primarily a case-study-style evaluation without quantitative clinical effect endpoints [19].

In intervention-oriented studies, QoC outcomes were frequently process and utilisation endpoints, such as serious-illness conversation rates [21], Advance-care planning (ACP) documentation rates [20], palliative care consultation rates [23] and service utilisation outcomes (e.g. readmissions, Intensive care unit (ICU) transfer and length of stay) [23]. In contrast, algorithm development and validation studies have emphasised discrimination performance metrics (e.g. area under the receiver operating characteristic curve (AUROC), sensitivity, specificity, accuracy and confusion metrics) [26–29, 31], and technology validation studies have reported agreement metrics (e.g. Bland–Altman, mean error, inter-rater reliability and validity coefficients) [25, 32]. Finally, studies evaluating clinician-facing systems used usability and expert judgment endpoints (e.g. System Usability Scale (SUS), Short User Experience Questionnaire – Short (UEQ-S) and expert ratings) [24, 33].

Additionally, some technology-focused studies have explicitly framed QoC benefits in terms of care processes. Grießhammer (2024) evaluated contactless monitoring to enhance routine symptom management, whereas Dong (2025) suggested that automated voice-PROM extraction could reduce the clinician documentation burden.

#### Quality of Care instruments identified include:

- Epic Systems electronic medical record ACP form entries (Epic, Verona, WI); National Quality Forum end-of-life quality measures (Gensheimer 2022) [20]
- Palliative care consultation; service utilisation outcomes (e.g. 30-, 60-, and 90-day readmission, ICU transfer and inpatient length of stay) (Wilson 2023) [23]
- Voice recognition rate (Dong 2024) [27]
- Predictive performance metrics (area under the curve, F1, precision, recall, specificity, sensitivity, accuracy and confusion metrics) (Kawashima 2024, Dong 2024, Yang 2021, Liu 2023, Nejatifar 2025) [26–29, 31]
- Expert Likert scale ratings of overall treatment proposal, evidence/guideline conformity and applicability of recommendations (Braun 2024) [24]
- SUS (Blanes-Selva 2023) [33]
- Short UEQ-S (Blanes-Selva 2023) [33]
- Rate of documented serious-illness conversations (Manz 2023) [21]
- Rate of ACP documentation (Gensheimer 2022) [20]
- Rate of palliative-care referral (Wilson 2023) [23]
- Inter-rater reliability/validity coefficients (correlation coefficient, ICC, Cohen's kappa, and Gwet's AC) (Sampson 2025) [32]
- Agreement Analysis (Bland–Altman plot, mean error) (Grießhammer 2024) [25]
- Minimal Documentation System for routine daily symptom assessment (Grießhammer 2024) [25]

### Health related outcomes

Across the 15 studies, AI applications demonstrated predominantly positive or neutral-to-positive effects on the measured outcomes with no clear negative effects reported (see Table 4). The strength of this inference varied according to the study type. Interventional studies tended to report clinically interpretable processes or utilisation endpoints [20–23], whereas algorithm development and validation studies largely report proxy technical metrics (e.g. discrimination, agreement and reliability) rather than downstream patient benefit [25–29, 31, 32]. When statistical testing was reported, results were often significant (*p* < 0.05), especially in intervention studies and some validations [20–23, 25, 30–32]. Several technical studies have described performance descriptively with limited or no hypothesis testing [24, 26, 28, 29]. Overall, the findings are promising, but at an early stage. Many studies have shown that this model can work rather than improving care.

**Table 4:** Effects of AI and direction of effect from the included studies

Study (first author, year)	Domain	Outcomes measured or discussed	Instrument/method	Direction of effect	Effect size	Statistical technique & result	Statistical significance	Comparison to usual care
Haleem, 2025 [30]	Quality of Life (QoL)/Quality of Care (QoC)	HRQoL	Multimodal deep-learning regression for estimating HRQoL from smartwatch data and PROMs	Positive	MAPE = 0.242	Linear regression, prediction error, Spearman correlation	No clinical effect test; Only *p* <.05/.01/.001 for Spearman correlation	No comparison to usual care
Kamdar, 2024 [22]	QoL	Pain (NRS), QoL domain scores	Digital therapeutic application (ePAL) for pain management vs control	Neutral–positive	Mean pain scores: 2.99 (ePAL) vs 4.05 (Control)	Descriptive statistics; two-sample t-tests, Chi-square, mixed linear effect models	*p* <.05; pain improved (significant); QoL domains did not differ	People receiving palliative care with ePAL were associated with lower pain and fewer pain-related hospitalisations
Blanes-Selva 2023 [33]	QoC	Usability & user experience of palliative clinical decision support systems (CDSS)	SUS and UEQ-S	Neutral–positive	SUS = 62.7 (Round 1)/65.0 (Round 2); UEQ-S = 1.42 (Round 1)/1.5 (Round 2)	Descriptive statistics; t-test	*p* <.05; round-to-round differences were not significant	No comparison to usual care
Bowles, 2024 [19]	QoC	Feasibility of AI-generated summaries for supporting palliative-care needs assessment	AI-generated narrative summaries from administrative claims data	Neutral–positive	N/A	Qualitative focus group analysis from three palliative-care team members	N/A	AI provides sufficient assessment for palliative-care needs but is not adequate for direct clinical recommendation
Yang, 2021 [28]	QoL	Survival outcomes via physical activity	LSTM network using wearable actigraphy	Neutral–positive	AUC = 0.7292	Classification metrics, comparison with QoL-related indicators (KPS/Palliative Prognostic Index (PPI)	N/A	The model underperforms KPS and PPI; however, it does not require clinician input
Kawashima, 2024 [26]	QoC	Predicting specialist palliative-care needs	XGBoost using EHR-derived variables, patient-reported pain, and QoL-related indicators	Positive	AUC = 0.89	Classification performance metrics	N/A	No comparison to usual care
Nejatifar, 2025 [31]	QoC	Frailty-based prediction of palliative-care needs in chronic obstructive pulmonary disease	Super Learner model using demographics and frailty conditions	Positive	AUC = 0.92	Classification performance metrics, chi-square and t-tests for relationships, logistic prediction for predictors	*p* < 0.001	No comparison to usual care
Manz, 2023 [21]	QoC	SIC rate; end-of-life (EOL) systemic therapy; EOL utilisation outcomes	Machine learning (ML) mortality prediction via EHR data; behavioural nudge to prompt SICs	Positive	SICs: aOR 2.09; Reduced EOL systemic therapy: aOR 0.25	Descriptive statistics, generalized estimating equations, subgroup interaction analysis	*p* < 0.001	Behavioural nudges to clinicians led to an increase in SICs and reduction in end-of-life systemic therapy
Gensheimer, 2022 [20]	QoC	ACP documentation frequency; EOL quality measures	Prognosis model to predict survival from EHR data; lay care coaches	Positive	ACP documentation: OR 13.7; Prognosis documentation: OR 9.9	Mixed-effects logistic regression	*p* < 0.001	Combining a prognosis model with care coaches increased ACP documentation
Wilson, 2023 [23]	QoC	Palliative care consultation; readmissions; ICU transfer; length of stay	ML CDSS tool via EHR data integrated into clinical workflow	Positive	Palliative care consultations: IRR 1.44; 60-day readmission: OR 0.75; 90-day readmission: OR 0.72	Bayesian estimation accounting for the stepped design; 95% Credible Intervals	N/A	Increased palliative care rate consultations and reduction in hospitalisation
Sampson, 2025 [32]	QoC	Reliability and validity of a pain assessment tool	PainChek for automated pain assessment from facial analysis, voice, movement, behaviour activity and body	Positive	IRR 0.714 (rest), 0.817 (post-movement); Internal consistency 0.755 (rest), 0.833 (post-movement); Concurrent validity with PAINAD: 0.528 (rest), 0.787 (post-movement); Convergent validity with SM_EOLD −0.555 (rest), −0.544 (post-movement)	Inter-rater analysis, internal consistency, concurrent validity; comparison with Abbey Pain Scale (APS), PAINAD and SM-EOLD	N/A	PainChek compares favourably with APS
Griebhammer, 2024 [25]	QoC	Feasibility of radar-based heart rate monitoring in palliative care	Continuous-wave radar units installed underneath the head section of the slatted frame for contactless monitoring	Neutral–Positive	Mean Difference: Arm I − 0.852 beats per minute (bpm), ARM II − 0.678 bpm	Concordance analysis; Bland–Altman; two one-sided test (TOST)	*p* < 0.001	Moderate agreement when compared with electrocardiogram
Dong 2024 [27]	QoC	Accuracy of patient-reported pain symptom detection	ML to extract patient-reported pain symptoms from transcribed patient interviews	Neutral–positive	Voice recognition rate 55.6%; F1 0.31–0.46 over five symptoms	Voice recognition rate; classification metrics; Kruskal–Wallis test	*p* = 0.515 for differences in recognition rate across disease groups	Moderate performance compared with human-transcribed Integrated Palliative care Outcome Scale
Liu, 2023 [29]	QoC	7-day mortality prediction	ML to predict 7-day mortality from clinical assessment and wearable data	Positive	AUC = 0.960	Classification metrics	N/A	No comparison to usual care
Braun, 2024 [24]	QoC	Expert rating of ChatGPT therapy suggestions	ChatGPT to provide therapy suggestions on 10 patient case vignettes	Neutral–positive	N/A	Descriptive statistics	N/A	No comparison to usual care

Outcome Measures: *ACP* Advance Care Planning, *BPI* Brief Pain Inventory (Short Form composite severity score), *HRQoL* Health-Related Quality of life, *KPS* Karnofsky Performance Status, *NRS* Numerical Rating Scale, *PAINAD* Pain Assessment in Advanced Dementia Scale, *PROMs* Patient-Reported Outcome Measures, *SIC* Serious Illness Conversation, *SUS* System Usability Scale, *UEQ-S* User Experience Questionnaire–Short, *SM-EOLD* Standardised Modified End-of-Life in Dementia

Instrument/Methodology: *EHR* Electronic Health Record, *LSTM* Long Short-Term Memory, *RCT* Randomised Controlled Trial, *XGBoost* Extreme Gradient Boosting

Statistical/Results Metrics: *ACC* Accuracy, *aOR* Adjusted Odds Ratio, *AUC* Area Under the Curve, *F1 score* Harmonic Mean of Precision and Recall, *IRR* Incidence Rate Ratio, *OR* Odds Ratio, *MAPE* Mean absolute percentage error

The evidence is clearest in studies that compared with usual care. Three RCTs showed superiority over usual care for clinically meaningful outcomes: improved serious-illness conversation documentation with reduced end-of-life systemic therapy [21], increased palliative-care consultation activity with fewer readmissions [23], and reduced pain intensity with fewer pain-related hospitalisations [22].

By contrast, algorithm development and validation studies typically report technical or procedural proxies. Prognostic and palliative care needs identification models frequently reported good-to-excellent AUROC values and high sensitivity [26, 29, 31]; however, these metrics were rarely linked to demonstrated changes in care delivery. Survival prediction is feasible with moderate prognostic accuracy or agreement relative to a standard functional status tool [28]. Technology validation studies emphasise agreement, accuracy and reliability against reference standards [25, 32], whereas clinician-facing systems emphasise usability and expert judgment endpoints rather than patient outcomes [24, 33].

### Practical and technical consequences of applying AI in palliative care

This section outlines the explicit practical and technical considerations raised by the authors of the 15 included studies. The implicit considerations, identified by the review team, are discussed under the discussion section. These practical and technical considerations were extracted, categorised and discussed within the team (TNP, a radio oncologist; TRP, a qualitative researcher and palliative care doctor; SC, a computer scientist and AI expert; KT, a computer scientist) along with implicit relevant ethical considerations identified. This process was iterative, ensuring that the clinical and logistical practicality, AI developmental and implementation phase as well as neglected ethical considerations were robustly captured.

Two documents were used as frameworks during this process: World Health Organization’s six governing principles for AI in health (2021) [10] and the UKRIO’s practical guide for AI researchers (2025) [14].

Ethical engagement specific to AI in the included studies was strikingly limited. Therefore, while explicit practical and technical considerations are described here, the fuller synthesis and mapping of ethical considerations against established frameworks is presented here in the Discussion.

### Practical considerations

Integration into existing clinical workflows is a major barrier. Implementation-oriented work explicitly highlighted the need to avoid creating additional burdens (e.g. adding extra electronic health record (EHR) alerts) [19, 21–23, 33], with one study specifically designing an intervention to abrogate the potential for alert fatigue by avoiding a best-practice alert [23]. Clinician-facing tools have also raised practical concerns about how outputs fit into daily work [19], and usability studies have emphasised the need for systems that support professional autonomy within real clinical workflows [33]. Overall, participants indicated that AI systems should not disrupt routine practice or require additional interpretation time from already overburdened palliative-care teams [19, 21–23, 33]. Palliative clinicians have also reported severe staffing shortages and “no spare capacity” to learn or troubleshoot new systems [19, 22].

Furthermore, wearable device studies highlight patient- and unknown-level burdens, including discomfort, device management, and data upload compliance [28–30]. In actigraphy-based survival prediction, people receiving palliative care were instructed to wear the device throughout admission, except during showering, because it was not water-resistant, and the data required periodic uploads [28]. In smartwatch-based mortality prediction, participants needed to operate smartphone syncing; some days’ data were excluded when uploads were absent, and user feedback reported charging problems and minor skin irritation [29].

### Technical considerations

Models rely heavily on structured EHRs or claims data of variable quality, suffer from class imbalances, and exhibit limited generalisability beyond their original institutions and condition cohorts [26, 31]. Moreover, class imbalance is common (e.g. a low prevalence of palliative care needs or mortality events) and is typically addressed by undersampling or ensemble methods, thereby reducing effective training data [26, 28].

Regarding generative AI, LLM-based approaches raise risks around incorrect or unsafe content generation and the need for careful human oversight [24], while narrative-summary approaches raised pragmatic concerns about the nature of outputs (e.g., length/fit-for-workflow and confidentiality) that affect real-world utility [19]. Deep-learning systems can remain difficult to interpret; however, some studies used explainability approaches such as SHapley Additive exPlanations (SHAP) analyses for erroneous predictions [29] and others incorporated “explanatory” design features intended to preserve clinicians’ professional autonomy [33].

## Discussion

This scoping review identified three primary domains of AI application in palliative care: (1) early identification of palliative care needs through prognostic modelling (*n* = 9), (2) symptom assessment and relief using sensor-based and multimodal tools (*n* = 6) and (3) clinical decision support for care conversations via nudges and generative models (*n* = 4), with some studies contributing to more than one domain. These align with broader trends in the field where predictive analytics have dominated early efforts, particularly from 2021–2023, reflecting the maturation of ML techniques for mortality forecasting and palliative care identification from EHRs and related structured data [20, 21, 23, 28, 29, 33, 36]. A bibliometric analysis reports of this shift reported that early AI research (pre-2023) focused on prognostication to address late referrals, with annual outputs rising from a few papers to a peak of 66 in 2024, driven by multimodal integration [37].

The surge in symptom assessment applications in 2024–2025 mirrored the rapid adoption of wearable and voice technologies following the COVID-19 pandemic. This is evident from a scoping review that highlighted the role of DL in real-time HRQoL estimation and pain detection in publications increasing since 2023 [13]. Nevertheless, decision support remains consistent, but niche, often leveraging LLMs such as ChatGPT for narrative generation [19, 24], which is consistent with the wider literature on generative AI's potential for communication aids [11].

Overall, the convergence across domains, from siloed prediction to integrated systems, parallels global trends toward "explainable AI," which emphasises hybrid models for end-of-life personalisation [7]. However, with most studies originating from Global North settings, these innovations risk irrelevance in low-resource contexts, where new technologies, such as low-cost radar monitoring [25], show promise but require Southern-led validation.

Regarding the effectiveness on QoL and QoC, AI demonstrated positive effects across the measured outcomes, with moderate to large effect sizes and consistent statistical significance. Three RCTs reported superiority to usual care in conversation initiation, referrals, and pain outcomes (Table 4).

Direct measurement of QoL is rare [22, 30], but indirect benefits via earlier interventions have been noted, aligning with the literature showing that AI-enhanced symptom management can improve global health scores on scales such as the EORTC Quality of Life Questionnaire Core-30 [22]. Wider evidence supports these findings. Reviews of ML in palliative care found that predictive tools reduced high-intensity end-of-life treatments, correlating with better QoL [7, 38], while bibliometric trend projects continued to grow in AI for personalised comfort [36].

Nevertheless, QoC outcomes dominated (100% of studies), with strong evidence of usability (SUS scores 62–65) (see Table 4) and process improvements (e.g. an 11% absolute referral increase) (see Table 4), echoing a 2025 systematic review that attributes such gains to routine data integration and enhanced resource allocation in understaffed settings [38]. The emphasis on QoC over QoL reflects a broader skew in the literature, which limits generalisability [13]. This QoC bias risks overlooking holistic palliative goals, where AI's true value lies in augmenting rather than just streamlining care.

### Practical considerations

The identified practical considerations include workflow disruption, alert fatigue, and resource demands, mirroring the wider challenges in AI implementation. Integration issues such as time burdens from AI outputs [19, 33] align with a recent review which reported that the implementation of palliative AI is stalled by clinician overload, particularly in underresourced contexts [7]. Furthermore, inherent barriers to wearable compliance [28, 30], namely high dropout rates due to discomfort in frail people receiving palliative care and usability, underscore the need for co-design [36]. Small, single-centre studies further constrain scalability, consistent with calls for interdisciplinary piloting to bridge the feasibility of practice [11, 36]. Moreover, many of the included studies were small, single-centre feasibility or validation projects with limited sample sizes, restricting generalisability and real-world scalability. Interdisciplinary co-design, iterative piloting, and dedicated staff training are essential prerequisites for acceptability; however, these elements are present in only a few studies [30, 33].

### Technical considerations

Regarding technical considerations, technical limitations such as data quality variability, class imbalance, and poor generalisability [26, 31] are recurrent patterns in the palliative AI literature. That is, overreliance on EHRs tends to lead to missing values, thereby reducing model accuracy [36]. Black-box issues persist despite SHAP’s attempts [33], mirroring the wider literature in which interpretability remains challenging [7]. Moreover, infrastructure requirements [19] and input length requirements [28] highlight scalability gaps to enhance robustness across sites [7, 13].

Data requirements can also be nontrivial; in wearable actigraphy survival prediction, performance was better when using 48-h of input data compared to 24-h [28], and wearable-based studies depended on sustained device use and data completeness (e.g. synchronisation and handling missingness) [29, 30]. Validation in several studies ends with technical performance metrics rather than sustained clinical monitoring and downstream outcomes, limiting its usefulness for implementation decisions.

### Ethical considerations and mitigation strategies

Ethical engagement specific to AI in the included studies was strikingly limited and was typically confined to baseline research governance, with bias, autonomy, and equity rarely addressed in depth. For instance, prospective wearable work reported Institutional Review Board approval and informed consent [28, 29]; however, explicit discussions of *AI-specific* consent, communication with participants and equity/fairness implications were generally limited in reporting across study types.

When mapped against the World Health Organization’s six governing principles for AI in health (2021) [10] and the UKRIO’s practical guide for AI researchers (2025) [14], the evidence reveals major gaps that must be addressed before widespread adoption in high-stakes, vulnerability-intensive fields such as palliative and end-of-life care:

#### Principle 1 – protecting human autonomy

Multiple studies have highlighted clinicians’ concerns that AI can diminish clinical judgement or the human elements of care [19, 33]. This concern is justified, as any perception that decisions are delegated to an algorithm risks undermining trust and the therapeutic relationship between HCPs and people receiving palliative care. Informed consent specifically for AI processing of data is rarely described; in wearable studies, this may be implied rather than explicitly stated.

##### Mitigation

Human-in-the-loop must be mandatory with explicit clinician override and explicit tiered consent for AI use, with people receiving palliative care retaining the right to opt for human-only care pathways. These strategies aim to preserve the doctor-patient relationship, which is the cornerstone of palliative care and ensure that AI remains a supportive tool rather than a replacement for human compassion and care.

#### Principle 2 – promoting human well-being, safety and the public interest

High-sensitivity predictive models risk causing unnecessary distress through false positives or premature palliative-care discussions if poorly governed [23, 26]. Although some studies have used firewalls and internal processing [19, 30], none have reported formal Data Protection Impact Assessments or compliance with the World Health Organization’s recommended minimum necessary data principles [10]. This is problematic because inadequate privacy safeguards risk re-identification of sensitive end-of-life data. Specifically, participants in wearable studies [28–30] were exposed to re-identification risks through continuous behavioural data.

##### Mitigation

Context-specific thresholds, continuous post-launch surveillance, rapid error reporting, data minimisation, pseudonymisation, and independent security audits should be implemented. These suggestions ensure that participants’ data privacy is safeguarded, and ongoing mechanisms are in place to report potential data breaches.

#### Principle 3 – ensuring transparency, explainability and intelligibility

Despite some explainability attempts (e.g. SHAP analyses) [29, 33], most studies deploy black-box models without patient-facing explanations or documentation of limitations. The black-box nature inherently limits HCPs’ trust in the modules and goes against people receiving palliative care’s right to understand how decisions affecting their care are made.

##### Mitigation

Patient-facing plain language explanations, validated explanation strategies (e.g. SHAP/Local Interpretable Model-agnostic Explanations where appropriate), and transparent documentation of the model’s intended use, limitations, and known failure modes.

These strategies ensure that people receiving palliative care and their families meaningfully engage in shared decision-making regarding their care.

#### Principle 4 – fostering responsibility and accountability

No study has reported a formal ethical review of AI components or established accountability mechanisms. It can be inferred that the relevant ethics committee approved the studies; however, AI-specific oversight could be further contested.

##### Mitigation

Palliative-specific AI governance committees with patient/family representation and public registration of health AI systems are required. These structures are necessary to ensure that accountability is shared among developers, clinicians, and institutions, thereby protecting vulnerable people receiving palliative care from unaddressed harm.

#### Principle 5 – ensuring inclusiveness and equity

Fourteen studies originated from high- or upper-middle-income settings with limited ethnic, socioeconomic, or geographic diversity, potentially widening the existing palliative care inequities in underserved populations. Certain study designs such as wearable-heavy approaches intentionally exclude people receiving palliative care with poor digital literacy or physical limitations [28–30], perpetuating digital exclusion in this vulnerable and seldom-heard-of group.

This geographic skew also raises concerns of epistemic injustice, including the suppression of difference through different linguistic features, cultural contexts, and resources. Data harvested mainly from the Global South is often used to feed and train models predominantly serving the Global North, contributing to epistemicide and hermeneutical erasure of non-Western epistemologies [39].

##### Mitigation

Bias audits with fairness metrics, diverse multinational training data, community co-design, and low-tech alternatives with accessibility testing are required, all of which are needed to prevent AI from exacerbating inequities in palliative care.

#### Principle 6 – promoting AI that is responsive and sustainable

None of the studies mentioned environmental concerns, and the long-term responsiveness and sustainability of wearable devices remain unaddressed. Unsustainable designs increase ecological burden and exclude people receiving palliative care in resource-constrained settings, contradicting the holistic ethos of palliative care.

##### Mitigation

Environmental impact assessments and designs that are viable across resource settings must be incorporated. This is appropriate, because palliative care should consider broader societal and planetary contexts.

##### Cross-cutting issue – Absence of informed consent for AI use (Principles 1, 2, and 3)

Retrospective data studies [26, 31] and wearable devices routinely fail to describe specific consent for AI processing. This could be implied as an ethics committee waiver for secondary analysis; however, this was not explicitly stated. Without explicit consent for AI use, participants are denied agency over the use of sensitive data, potentially violating their autonomy. This includes protecting privacy, confidentiality, and limiting the use of people receiving palliative care’s data for AI training beyond what is necessary, as well as respecting individual uniqueness beyond aggregated training data. There are also implications of AI systems potentially, and selectively, incorporating data from published studies, for which privacy-preserving approaches should be considered where feasible [40].

Moreover, identified ethical considerations such as bias from homogeneous data, unclear AI consent, and opacity can amplify vulnerabilities in palliative care research and delivery. Given that most of the included studies (93%) were from Global North settings, this skewed data underperforms in diverse populations [10].

These ethical concerns can be addressed by embedding relevant AI ethical frameworks, such as those from the World Health Organization or UKRIO, to ensure that AI use by participants is appropriately safeguarded [10, 14].

##### Mitigation

Explicit, tiered consent or valid ethics-committee waiver is mandatory. Future studies should consider use of privacy-preserving or non-training AI tools, where possible.

Table 5 summarises the identified ethical considerations and proposed mitigation strategies.

**Table 5:** Identified ethical considerations and proposed mitigation strategies

Ethical considerations	WHO (2021) Principle	Mitigation strategies
Bias, discrimination, marginalisation and digital exclusion from: - Homogeneous datasets - Wearable/application barriers - Developer biases - Bias in deployment	5 – Inclusiveness and equity 6 – Promote artificial intelligence that is responsive and sustainable	- Bias audits and fairness metrics; diverse multinational data; community co-design - Protecting/minimising data colonialism - Early and ongoing stakeholder engagement - Low-tech alternatives - Accessibility testing with seldom-heard-of populations
Inadequate privacy/data protection	2 – Promote human well-being, human safety and the public interest	- Mandatory data protection impact assessment - Data minimisation - Pseudonymisation - Independent security audit
Over-reliance on artificial intelligence (AI), erosion of autonomy and potential absence of informed consent for AI use	1 – Protect autonomy 6 – Promote AI that is responsive and sustainable	- Human-in-the-loop as mandatory mechanism - ‘Graceful transition’ period and training for staff - Explicit tiered consent for AI processing - Right to opt for human-only pathway
Lack of transparency and explainability	3 – Ensure transparency, explainability and intelligibility	- Patient-facing explanations - publish model cards - Validated explainability modules - Regular AI Audit
Harm from false positives/negatives	2 – Promote human well-being, human safety and the public interest	- Context-specific thresholds - Continuous AI performance surveillance - Rapid-error reporting process
No formal governance or accountability	4 – Foster responsibility and accountability 6 – Promote AI that is responsive and sustainable	- Palliative-specific AI governance committee; public registration of systems - Impact assessment at protocol stage

Overall, this scoping review maps AI's evolving role of AI in palliative care, but reveals an immature, northern-centric evidence base. Future work must prioritise diverse participatory trials to realise equitable benefits guided by more robust AI ethical frameworks

### Strengths of the study

We included a wide range of study designs to provide a comprehensive overview of real-world data on AI applications in palliative care settings. The methodology was rigorous with a prospectively registered protocol, dual-independent screening, and structured data extraction with verification at both the title/abstract and full-text stages, along with regular team discussions for robust interpretation.

The review team was deliberately diverse and comprised two palliative care clinicians/researchers and two computer engineers with AI expertise. Three members are based in the Global South (Thailand) and one is in the Global North, offering a more balanced perspective on the opportunities and equity implications of this emerging technology in different resource contexts.

We also identified equity, diversity, and inclusion issues, including the digital divide, limited access to technology in low-resource settings, and the risk that most models are trained predominantly on data from high-income, white-majority populations, which warrant urgent further exploration in future research to prevent data colonisation [10].

Finally, we systematically outlined practical, technical and ethical considerations against established frameworks [10, 14], thus providing a clear roadmap for responsible AI development in palliative care.

### Limitations

This review has several limitations:

- First, the searches were limited to studies published in English and in five major databases; relevant research in other languages or unpublished/grey literature may have been missed.
- Second, consistent with the JBI scoping review guidelines, no formal critical appraisal of methodological quality or risk of bias was performed; therefore, readers should interpret effectiveness claims cautiously, particularly given the predominance of small, single-centre studies. Moreover, no meta-analysis was conducted owing to substantial heterogeneity in study designs, AI modalities, and outcome measures, which is an expected limitation of scoping reviews and is fully in line with JBI recommendations.
- Third, the evidence is heavily skewed toward Global North institutions (93%), restricting insights into their applicability in low- and middle-income settings.

Finally, studies with negative or null results were frequently underreported [38]. For the quantitative and mixed-methods studies included here, publication bias may have favoured more positive findings, potentially influencing our overall interpretation. However, we have remained impartial and reported the data exactly as presented by the original authors. A practical suggestion beyond this review is to encourage routine publication or registration of all trial results [38], including negative findings, as these provide invaluable insights for the safe development of AI in palliative care.

### Implications for future studies

Additional qualitative and co-designed studies are required. Only one included study used a qualitative method, and the voice of people receiving palliative care and their caregivers were almost entirely absent beyond providing physiological or voice data. Even in studies using voice recordings [27] or wearables [28–30], people receiving palliative care contributed physiological or conversational data but had no clearly documented role in tool design, threshold setting or interpretation of outputs. Therefore, most AI interventions remain clinician-centred, prioritising HCP workflow efficiency and clinical data extraction over direct patient agency.

In-depth interviews, focus groups, or experience-based co-design approaches could provide richer insights into how people receiving palliative care, families, and clinicians perceive and interact with AI tools in the emotionally charged context of palliative care. Such studies would help ensure that future technologies remain person-centred rather than clinician- or data-driven [41].

Mixed-methods designs should be prioritised to capture both measurable effectiveness (e.g. referral rates and prognostic accuracy) and contextual factors, such as cultural acceptability, trust, and relational impact. This strengthens the evidence base and improves real-world applicability.

There is an urgent need to improve geographic diversity. That is, fourteen of the fifteen studies originated in the Global North, with only one from Iran representing the Global South, demonstrating potential epistemic inequality. This imbalance limits our understanding of how cultural norms, varying levels of digital literacy, health-system infrastructure, and resource constraints shape AI implementation. Future research should actively involve partners and study sites from low- and middle-income countries to produce more contextually grounded, equitable AI solutions for palliative care worldwide.

Future studies should also prioritise robust data governance to protect participants’ data post-study and prevent further unauthorised harvest by AI systems. Given the sensitive nature of palliative care data and the risk that published datasets or study outputs could be scraped and incorporated into large language models or other AI training corpora without explicit ongoing consent, researchers must implement clear data use agreements, time-limited access, and technical safeguards such as differential privacy or data deletion protocols. This is essential to uphold person-centred care principles and prevent exploitation of vulnerable participants’ information beyond the original study purposes.

## Conclusions

AI demonstrates clear potential to enhance palliative care by enabling earlier identification of needs, supporting real-time symptom assessments, and facilitating decision-making and care conversations. The 15 studies in this review showed generally favourable findings in prognostic accuracy, referral rates, conversation initiation, and pain management compared to that of usual care, with emerging evidence of positive effects on HRQoL. This field is rapidly maturing and moving from isolated predictive models to integrated multimodal systems. However, the current evidence base remains small, predominantly from Global North settings, and is limited in its focus on patient-reported outcomes and ethical governance.

To translate these promises into an equitable, patient-centred reality, future research should prioritise larger, geographically diverse studies with explicit HRQoL endpoints, qualitative and co-design approaches that centre patient and caregiver experience, active collaboration with Global South partners, and systematic application of AI ethical frameworks from the outset. When responsibly developed, AI may significantly strengthen the compassionate and holistic delivery of palliative care.

## Supplementary Information

Supplementary Material 1.

Supplementary Material 2.

## Data Availability

All data generated or analysed during this study are included in this published article and its supplementary information files.

## Abbreviations

ACP: Advance-care planning
AI: Artificial Intelligence
AUROC: Area Under the Receiver Operating Characteristic curve
CDSS: Clinical Decision Support Systems
COPD: Chronic Obstructive Pulmonary Disease
DL: Deep Learning
EHR: Electronic Health Record
GBM: Gradient Boosting Machine
HCPs: Health Care Professionals
HRQoL: Health-Related Quality of Life
ICU: Intensive care unit
IPOS: Integrated Palliative Care Outcome Scale
JBI: Joanna Briggs Institute
KPS: Karnofsky Performance Status
LLM: Large Language Model
LSTM: Long Short-Term Memory
ML: Machine Learning
NLP: Natural Language Processing
NRS: Pain Numerical Rating Scale
PROM: Patient-Reported Outcome Measures
QoC: Quality of Care
QoL: Quality-of-Life
RCT: Randomized Controlled Trial
SHAP: SHapley Additive exPlanations
SUS: System Usability Scale
UEQ-S: User Experience Questionnaire- Short
UKRIO: United Kingdom Research Integrity Office
WHO: World Health Organization
XGBoost: Extreme Gradient Boosting
