# Supplementary material for: Exploring the application of Artificial Intelligence in palliative care and its practical, technical and ethical considerations: a scoping review
Source: BMC Palliat Care. 2026 Jun 29;25:270. doi: 10.1186/s12904-026-02191-0 (PMC13573398; doi:10.1186/s12904-026-02191-0)
Supplement: Supplementary file 1 [file 12904_2026_2191_MOESM1_ESM.docx]

**Supplementary file 1. Search terms**

| **Database** | **Search terms** | **Date** | **Numbers of articles** |
| --- | --- | --- | --- |
| CINAHL Ultimate | (palliative care or terminal care or end of life care) AND (artificial intelligence or ai or a.i.) | 19.9.25 | 174 |
| PubMed | ((Artificial Intelligence[MeSH Terms]) OR (Artificial intelligence[Title/Abstract])) AND (((Palliative care[MeSH Terms]) OR (Hospice and Palliative Care Nursing[MeSH Terms])) OR (Palliative care[Title/Abstract] OR end-of-life care[Title/Abstract] OR terminal care[Title/Abstract])) | 25.9.25 | 282 |
| CENTRAL |  | 22.9.25 | 25 |
| ACM | [Abstract: "artificial intelligence"] AND [Abstract: palliative] | 25.9.25 | 84 |
| Web of Sciences | "artificial intelligence" (Topic) and Palliative care OR end-of-life care OR terminal care (Topic) | 25.9.25 | 249 |
