# Supplementary material for: Exploring the application of Artificial Intelligence in palliative care and its practical, technical and ethical considerations: a scoping review
Source: BMC Palliat Care. 2026 Jun 29;25:270. doi: 10.1186/s12904-026-02191-0 (PMC13573398; doi:10.1186/s12904-026-02191-0)
Supplement: Supplementary file 2 [file 12904_2026_2191_MOESM2_ESM.docx]

**Supplementary file 2. AI development process**

| **Article** | **Name of AI** | **AI type** | **Input** | **Output** | **Model detail** | **Development process** | **Validation process** | **Testing process** | **Performance** |
| --- | --- | --- | --- | --- | --- | --- | --- | --- | --- |
| (Bowles et al., 2024) | AI Chatbot with a prompt template and sample output. | Generative AI / Large Language Model (LLM) integrated with a 12-month mortality risk algorithm incorporating ~3,500 predictors from medical and pharmaceutical claims data and clinician-identified triggers. Translating structured data into narrative summaries (“Fact Sheet”). | Output data from the 12-month mortality prediction algorithm (features from claims data, SHAP values, and model explanations). Prompt template describing patient risk, health profile, and care recommendations. | Narrative summary in clinical-note format including: (1) Risk of mortality, (2) Patient health profile, (3) Implications and recommended next steps for patient care. | Open-source generative AI model (LLM) runs on internal GPU servers (4 GPUs) behind VNS Health’s firewall using a custom chat bot interface to ensure data security. | Collaboration between data scientists and palliative care clinicians to co-design the “Fact Sheet” template; iterative prompting with sample outputs; internal deployment for clinician testing. | Informal validation via clinician feedback assessing usefulness, clarity, and accuracy of AI-generated summaries. No quantitative performance validation reported. | Pilot testing on 3 patient cases identified by the mortality algorithm; feedback collected from 3 palliative care clinicians. Data processed internally to maintain confidentiality and data protection. | Clinicians found summaries helpful but too long and general. AI output was considered promising but required refinement for clarity, specificity, and clinical adequacy.  Challenges noted: AI hallucinations, misinformation, overgeneralization, and need for staff training. |
| (Gensheimer et al., 2023) | Machine Learning Survival Prediction Model (Stanford EMR-based model) | Supervised Machine Learning (predictive model for survival time) | High-dimensional Electronic Medical Record (EMR) data — including provider notes, radiology reports, laboratory results, vital signs, and procedure codes | Predicted median survival time (binned into 0–6, 6–12, 12–24, and >24 months) | Ensemble machine learning model (type not explicitly specified in this paper; developed previously by Gensheimer et al., J Natl Cancer Inst 2019) trained on metastatic cancer EMR data | Developed previously at Stanford University using thousands of EMR variables to predict life expectancy for metastatic cancer patients; trained and internally validated before this study | Previously validated in independent datasets; shown to outperform traditional prognostic models and match physician accuracy in survival prediction (referenced prior publications) | Integrated into clinical workflow and prospectively applied in this quality improvement project (June 2020–Feb 2021) to identify high-risk patients (< 2-year predicted survival) | Reported as reliable; prior validation showed better performance than traditional models and equivalent to treating physician accuracy (specific AUC not reported in this paper) |
| (Manz et al., 2023) | Machine learning 180-day mortality prediction algorithm | Machine learning mortality prediction algorithm | Validated electronic health record (EHR)–based model predicting 6‑month mortality. | Patients with ≥10% predicted mortality risk were flagged as “high risk.” Algorithm outputs were used to trigger behavioral nudges to clinicians. | Described only as a “prospectively validated machine learning algorithm to predict 6-month mortality”; no algorithm family (e.g., GBM, RF) or feature set is specified in this article and details are referred to a prior validation study | Not described in detail in this trial report. The model was developed and prospectively validated before this study; here it is treated as an already-built ML model integrated into the EHR and used to generate mortality risk estimates | The paper states that a “prospectively validated machine learning algorithm predicting 6-month mortality” was used, and that patients with ≥10% absolute mortality risk were deemed high risk. Performance and validation dataset details are not re-reported here (they are referenced to Manz et al., JAMA Oncol 2020). | Real-world deployment within 9 tertiary/community oncology clinics in a stepped-wedge randomized trial: the algorithm ran within the health system’s EHR to identify high-risk patients, generate weekly lists of ≥6 forthcoming high-risk encounters, and trigger opt-out text reminders to clinicians before those visits. | Mortality model metrics (e.g., AUROC, calibration) are not reported in this paper. Instead, the article reports downstream clinical outcomes: the intervention increased serious illness conversations (SICs) (e.g., high-risk encounters 3.4% to 13.5%) and reduced systemic therapy within 14 days of death (10.4% to 7.5%). |
| (Kamdar et al., 2024) | ePAL (Digital Therapeutic App) | Rule-based + Machine Learning (ML-assisted) triage algorithm — uses branching logic and simple AI decision support for symptom triage | ePAL prompted participants to enter their average pain score on a scale of 0−10 three times weekly during an eight-week period. -Participants can report symptoms without prompts at any point -pain score 0-3, no action -4-7, AI algorithm used with branching logic to determine the patient’s barrier to pain control based on participant responses to a series of app-prompted questions about their symptoms. -8-10, PC triage nurse notified to call participants within an hour -Participants received daily coaching tips, which were tailored through ePAL’s AI platform to address recent barriers to cancer pain management. | Categorization of pain severity: 0–3 no action, 4–7 AI identifies barriers to pain control and triggers patient education module or clinician alert, 8–10 immediate nurse triage call within 1 hour - Tailored daily coaching tips generated via AI to address recent pain management barriers | Embedded triage algorithm within the ePAL app using branching logic rules (not a deep learning model); automates real-time decision support and education triggering | By software engineers at Partners Connected Health Innovation and the MGH Division of Palliative Care | Pilot-tested internally at MGH before trial; face validity through patient and clinician feedback during app refinement (no external or algorithmic validation reported) | Evaluated through a randomized controlled trial (RCT) (N = 112; 56 intervention, 56 control) comparing ePAL + usual care vs usual care alone | ePAL reduced mean pain scores significantly (Week 8: 2.99 vs 4.05, p = 0.017) - 69% reduction in pain-related hospitalizations (7.1% vs 23.2%, p = 0.018) - Improved barriers-to-pain management (B = –0.04, p = 0.042) - Mild increase in anxiety (B = 0.21, p = 0.015) - Adherence declined from 86% to 48% weekly users by Week 8 |
| (Wilson et al., 2023) | Control Tower / Acute Care Multi-patient System (AMP) | Supervised Machine Learning through Gradient Boosting Machine (GBM) | EMR-derived features: demographics, prior hospitalizations, comorbidities, labs, time-varying data, hospitalization events. | Risk score (0–100) interpreted as 7-day probability of needing palliative care; color tiers (Red ≥7, Yellow 4–6, White <4); ranked patient lists and top predictors. | GBM modeling a heterogeneous Poisson process (time-to-palliative consult → converted to 7-day probability). References Murphee et all for full technical details | Developed and previously described (Murphree et al.) | Developed and previously described (Murphree et al.) | Model ran live in institutional Control Tower; CTO generated daily top-risk lists (top 12/day) Mon–Fri 08:00–17:00 for clinician review during trial. | Operational metrics: initial reviewer–model agreement = 30.9%; average reviewer acceptance rate ≈ 20% (Q1 8%, Q3 31%); final agreement 38.9%. |
| (Braun et al., 2024) | LLM – ChatGPT (GPT-3.5, March 23, 2023 version) | Large Language Model (LLM) / Generative Pre-trained Transformer developed by OpenAI | Short case vignettes describing 10 simulated gynecologic oncology patients in a palliative setting. Each vignette was used to generate a patient-like prompt following the pattern: “I am an (age) year-old patient with (tumor diagnosis) (with metastases) and (symptom) in a palliative treatment situation. What therapy is available for my (symptom)?” The search history was cleared before each query. | Text-based therapy recommendations for each symptom — including drug and non-drug options — phrased in a polite, empathetic tone, and consistently advising users to consult a physician. | - Autoregressive transformer-based LLM (GPT-3.5). - Demonstrated pseudo-empathy (“I’m sorry to hear...”) and polite communication style. - Ethical design consideration: used only fictitious vignettes to avoid sharing sensitive patient data. | Developed by OpenAI as a general-purpose LLM trained on large-scale internet text. The study applied it as-is (no fine-tuning). Human experts provided oversight (“human-in-the-loop”) during evaluation. | No internal model validation performed by authors. Validation was conducted through expert evaluation of 10 generated responses for guideline conformity, applicability, and quality (Likert-scale rating). | In-silico testing: 10 simulated patient prompts were submitted to ChatGPT; outputs were independently assessed by five medical experts (3 gynecologic oncologists, 2 palliative care specialists). | Average expert rating: Overall quality = 4.1 / 5; Guideline conformity = 4.0 / 5; Applicability = 3.3 / 5. |
| (Grießhammer et al., 2024) | Continuous-wave radar units (iSYS-4001, InnoSenT GmbH, Donnersdorf, Germany) with embedded signal processing using a Hidden Semi-Markov Model (HSMM) algorithm | Machine-learning-based physiological signal processing (Hidden Semi-Markov Model, HSMM) | Raw displacement signals from 24 GHz radar sensors placed beneath hospital beds; data capture micro-movements of chest wall caused by heart sounds (S1, S2) and respiration | Extracted heart rate (HR) values and inter-beat intervals (IBIs); HR trends correlated with symptom burden and medication effects | HSMM algorithm detects first and second heart sounds from filtered radar displacement signals; computes IBIs and mean HR per interval; MATLAB-based custom script used for data processing | Developed based on prior radar-vital-sign studies (Will et al., 2018; Shi et al., 2019); implemented as a customized MATLAB script; algorithm parameters adapted for palliative-care patient data; training details not reported in this study | Compared radar-derived HR with gold-standard ECG from Holter ECG (Arm I) and Task Force® Monitor ECG (Arm II); equivalence confirmed using Bland–Altman and two-one-sided tests (TOST, p < 0.001) | Conducted on palliative-care inpatients (n = 30 Arm I, n = 13 Arm II); overnight and 1-hour recordings; results compared beat-to-beat or per 5-minute intervals; performance evaluated quantitatively and visually | High concordance with ECG: 92.7 % (Arm I) and 93.9 % (Arm II) within ±5 bpm; mean HR difference ≈ −0.8 bpm; strong correlation (r ≈ 0.93); equivalence statistically confirmed |
| (Kawashima et al., 2024) | ALL-model | Machine learning (XGBoost) | 126 variables including demographics, lab results, symptoms, and distress screening data | Binary prediction of specialist palliative care (SPC) need (Yes/No) | Gradient boosting trees with L1/L2 regularization using XGBoost | Trained on 80% of dataset with 5-fold cross-validation and grid-search hyperparameter tuning | Internal validation via 5-fold cross-validation within training data | Evaluated on 20% held-out test data; compared performance metrics (AUC, sensitivity, specificity) | AUC = 0.89; Sensitivity = 95.8%; Specificity = 71.9% |
| (Dong et al., 2025) | A voice recognition tool, “Ami Voice Medical Conference” | Speech-to-text (automatic voice recognition system using medical and general dictionaries) | Recorded conversational voice between patients and healthcare providers (Japanese language) - Environmental factors: background noise, distance from tablet, and patient positioning affected audio quality | Transcribed text of clinical conversations (speech converted into text) | Proprietary commercial speech recognition system optimized for medical meetings; not specifically trained for elderly or home-visit conversations | Used existing voice recognition system; recordings made via iPad Air 2 in home/nursing home settings; manually transcribed text used as gold standard for comparison | Accuracy validated by comparing automatically transcribed text with human-transcribed text; recognition rate calculated as correct words / total words | Tested across 100 patient interviews (2300 utterances); recognition rate stratified by disease type; lowest for stroke patients (48.3%); recognition rate affected by aging voices | Overall recognition rate = 76.1% (patients = 55.6%, healthcare providers = 82.2%) |
|  | Vertex AI (Google Cloud AutoML for text classification) | AutoML-based text classification (binary classification per symptom) | Automatically transcribed text data from Ami Voice - Labels derived from IPOS scores (positive ≥2, negative <2) | Predicted presence or absence of symptoms (e.g., pain, anxiety, depression, spiritual, social distress) | AutoML text classification with automatic preprocessing, model selection, and hyperparameter tuning; sensitivity adjusted to ~80% | Training: 40%, Validation: 20%, Testing: 40%; developed using Google Vertex AI on labeled text data | Performance validated using F1 score, precision, recall; best model selected based on validation data | Model tested on held out 40% dataset; performance metrics averaged across three repeated runs; ensured ~80% recall; | F1 scores across 5 symptoms = 0.31–0.46 Sensitivity (recall) = 0.39–0.75 Precision = 0.24–0.59 |
| (Yang et al., 2021) | Wearable actigraphy devices + survival prediction model | Deep Learning (specifically, Long Short-Term Memory Network or LSTM). LSTM is a particular type of Recurrent Neural Network (RNN) designed to handle time series data. | Three-dimensional actigraphy data: physical activity, angle, and spin, collected every second by wearable wristband (XB40ACT) during the first 24–48 hours of hospital admission | The prediction of the clinical status of patients at discharge, presented as a binary result: death (1) or discharged in stable condition (0). | One LSTM layer + TimeDistributed dense layer + Flatten + Dense layer; parameters adjusted (64 → 256 LSTM units, batch size 8–16, epochs 50–100, optimizer: Adam, loss: MAE) | Pre-processing involved zero-padding to equalize data length and averaging every 20 timesteps to reduce sequences to under 500 steps, preventing vanishing gradients. | The final model used 44 samples split into training, validation, and testing sets to prevent overfitting, trained for 100 epochs, achieving 0.97 training and 0.75 validation accuracy. | The final model used a 7:2:1 data split with six participants in testing, and performance was evaluated using sensitivity, specificity, PPV, NPV, and AUC. | The final model achieved 0.83 accuracy (AUC = 0.83, sensitivity = 1.0, specificity = 0.67), outperforming the 24-hour model and matching the prognostic accuracy of the KPS score. |
| (Liu et al., 2023) | Smartwatch, Garmin -ML | Supervised Machine Learning (ML) and Deep Learning (DNN) | Combined wearable data (steps, heart rate, SpO₂, sleep) + clinical assessments (appetite, urination, AKPS, care phase, etc.) + demographics (age, sex) | Binary prediction: 7-day death event (yes/no) among terminal cancer patients | Compared multiple models — Logistic Regression, SVM, Decision Tree, Random Forest, KNN, AdaBoost, XGBoost, and Deep Neural Network (MLP with hidden layers 64×3, ReLU activation). Feature differences from recent averages included to capture patient status changes. | Training-validation split (75%) and testing split (25%), stratified by label and sex. Positive events upsampled. Feature selection via ANOVA (SelectKBest). Hyperparameter tuning with grid search and 3-fold cross-validation. | 3-fold cross-validation within training-validation set; hyperparameters optimized and model retrained on full training-validation data. | Independent testing set (25%) used for model evaluation; performance metrics averaged over 100 random runs to reduce randomness. | XGBoost best model: AUROC = 0.96, F1 = 0.785, Accuracy = 0.93, Specificity = 0.97. DNN: AUROC = 0.933, F1 = 0.768. |
| (Haleem et al., 2025) | Multimodal Deep Learning Model for HRQoL Estimation | Deep learning (Multimodal regression model integrating CNN, BiLSTM, and Attention mechanism) | Physiological data from wearable devices (heart rate, sleep, stress, physical activity) and PROMs (HADS, IPOS, EORTC HRQoL-C30) | Predicted Health-Related Quality of Life (HRQoL) score | Parallel pipelines: CNN + BiLSTM + Attention for wearable (PHR) data and BiLSTM + CNN for PROMs data; outputs fused through dense layers with sigmoid activation | Implemented using Python and deep learning frameworks; trained on PASYKAF dataset (n = 204) with multimodal feature fusion; developed within GATEKEEPER Big Data platform | 5-fold patient-wise cross-validation to ensure independence of samples and reduce bias | Evaluated on unseen folds; metrics included MAE, RMSE, and MAPE for HRQoL prediction | Mean Absolute Percentage Error (MAPE) = 0.242; Mean Absolute Error (MAE) = 0.127; strong predictive accuracy for HRQoL trajectories |
| (Nejatifar et al., 2025) | Super Learner (Ensemble Model) | Ensemble meta-learning algorithm combining multiple base models | Predictions from all base models (Bayesian GLM, Random Forest, GAM, SVM, BART, Kernel k-NN, GLM) + original frailty and demographic variables | Final predicted need for palliative care (Yes/No) | Uses a Generalized Linear Model (GLM) as a meta-learner to optimally weight and combine base model outputs; runs iterative optimization until cross-validated risk improvement < 0.001 | Base models trained on 80% of data; Super Learner trained on stacked predictions; missing data imputed; iterative optimization for best weighted ensemble | 10-fold cross-validation to derive optimal weights and prevent overfitting | Evaluated on 20% hold-out test set using ROC curve, AUC, sensitivity, specificity, and F1 score | AUC = 0.92, Sensitivity = 91.46%, Specificity = 89.47%, F1 = 0.983 (highest among all models) |
| (Sampson et al., 2025) | PainChek | Artificial Intelligence (AI) combined with facial analysis and smartphone technology. It uses AI-powered algorithms. The function relies on the automated recognition of facial action units (AUs) indicative of pain. | A 3-second scan of the participant’s face using an app on a smart device (Apple iPad mini-4). The analysis focuses on recognizing specific Facial Action Units (AUs). | Total Pain Score (maximum score 42) and Severity Score (categorized as none, mild, moderate, or severe pain). | Architecture: A point-of-care smart device application. It utilizes automated recognition of facial pain signs. The assessment involves six domains, with Domain One being the automated AI facial assessment (measuring nine facial features of pain). The remaining five domains (Voice, Movement, Behaviour, Activity, and Body) are manually scored by the rater. | The AI uses algorithms to identify pain-relevant facial action units (AUs), which include tightening eyelids, nose wrinkling, and lips parting. The specific internal AI training methodology (e.g., dataset, epochs) is not detailed in the source material. | The current study performs a psychometric evaluation of PainChek in a general hospital setting. This involved assessing: Inter-rater reliability (IRR), Internal consistency (using Cronbach's alpha), Concurrent validity (against PAINAD and APS), Convergent validity (against SM-EOLD), and Discriminant validity (comparing rest vs. post-movement scores). | Data were collected by two independent raters (A and B) simultaneously. A total of 216 assessments were conducted. Sensitivity analyses were performed by removing six items that require 7 days of prior knowledge of the patient to see if this improved performance in the hospital setting. | Reliability: Showed substantial agreement between raters at both rest (0.714) and post-movement (0.817) (Correlation coefficient). Internal Consistency was good: 0.755 (rest) and 0.833 (post-movement) (Cronbach’s alpha). Validity: Showed moderate correlation with PAINAD at rest (0.528) and strong correlation post-movement (0.787). The model demonstrated significant discriminant validity (meaning scores increased significantly after movement provocation). |
| (Blanes-Selva et al., 2023) | The Aleph PC – Mortality Prediction Model | Machine learning–based classification model for 1-year mortality prediction | Administrative data, Barthel index, Charlson comorbidity index, laboratory test results, and diagnostic variables | Binary output: “High risk of 1-year mortality (Yes/No)” | Supervised ML model; deterministic once trained; includes SHAP explainability graph to visualize feature importance | Developed as part of The Aleph PC CDSS, an open-access ML web platform; designed through user-centred design involving clinicians; includes 3 main screens for data input, prediction output, and file management | Compared model predictions vs clinicians’ predictions on six real patient cases; evaluated using accuracy, sensitivity, and specificity | Tested in two rounds of usability and UX sessions with 21 healthcare professionals from six countries using the think-aloud method | Accuracy = 0.83, Sensitivity = 0.75, Specificity = 1.00 |
|  | The Aleph PC – Frailty Prediction Model | Machine learning–based classification model for 1-year frailty prediction | Administrative information, Barthel index, Charlson comorbidity index, laboratory test results, and diagnostic variables | Binary output: “Likely to become frail within 1 year (Yes/No)” | Supervised ML classification model using hospital admission data; deterministic predictions; includes SHAP explainability graph for transparency | Developed within The Aleph PC CDSS platform as part of an open-access ML system; follows user-centred design with clinician input during iterative interface development | Compared model predictions vs healthcare professionals’ predictions on six patient cases; evaluated with accuracy, sensitivity, and specificity | Evaluated in two usability and UX test rounds using the think-aloud method with 21 healthcare professionals from six countries | Accuracy = 1.00, Sensitivity = 1.00, Specificity = 1.00 |
|  | The Aleph PC – Mortality Regression Model | Machine learning–based regression model predicting expected survival time (in months) | Administrative information, Barthel index, Charlson comorbidity index, laboratory test results, and diagnostic variables | Continuous numerical output: Predicted survival time (months) | Regression-based supervised ML model built from hospital admission data; deterministic once trained; uses SHAP explainability graph to interpret feature influence on predicted survival duration | Developed as part of The Aleph PC CDSS platform; integrated within the same web-based interface as other models; data manually entered by users | Compared regression outputs (predicted months) with clinicians’ own interval-based predictions on six cases; performance evaluated by interval accuracy | Included in two rounds of usability and UX evaluations with 21 healthcare professionals using think-aloud sessions and satisfaction questionnaires | Accuracy = 0.67 (based on whether the real survival time fell within predicted interval ±2 months) |
